# LOXL1-AS1 predicts poor prognosis and promotes cell proliferation, migration, and invasion in osteosarcoma

**DOI:** 10.1042/BSR20190447

**Published:** 2019-04-30

**Authors:** Si Chen, Weiguo Li, Ai Guo

**Affiliations:** 1 Department of Orthopaedics, Beijing Friendship Hospital, Capital Medical University, Beijing 100050, China; 2 Department of Pain, Tongliang Hospital of Traditional Chinese Medicine Affiliated to Chinese Medicine of Chongqing Medical University, Chongqin 402560, China

**Keywords:** biomarker, LncRNA, LOXL1-AS1, osteosarcoma

## Abstract

lncRNA LOXL1 antisense RNA 1 (lncRNA LOXL1-AS1) was recently found to function as oncogenic lncRNA in glioblastoma, prostate cancer, and medulloblastoma. The role of LOXL1-AS1 in osteosarcoma was still unknown. In our study, we found LOXL1-AS1 expression levels were higher in osteosarcoma tissues and cell lines than normal bone tissues and normal osteoblast cell line, respectively. Moreover, high-expression of LOXL1-AS1 was correlated with Enneking stage, tumor size, distant metastasis, histological grade, and overall survival time in osteosarcoma patients. Furthermore, LOXL1-AS1 overexpression acted as an independent poor predictor for overall survival in osteosarcoma patients. The loss-of-function studies showed knockdown of LOXL1-AS1 dramatically inhibited osteosarcoma cell proliferation, migration, and invasion through suppressing PI3K-AKT pathway. In conclusion, LOXL1-AS1 predicts clinical progression and poor prognosis in osteosarcoma patients and functions as oncogenic lncRNA to regulate cell proliferation, cell cycle, migration, and invasion.

## Introduction

Osteosarcoma is one of the most common malignant bone tumors in children and young adults and accounting for 20% of all primary bone sarcomas [1,2]. Osteosarcoma is derived from bone marrow mesenchymal cells and limited to the metaphysis of long bones [3,4]. Due to rapid growth and early metastasis, at least one of every four osteosarcoma patients had distant metastasis losing operation opportunity at the time of initial diagnosis [5]. Although a multidisciplinary approach of surgery combined with chemotherapy significantly improve the 5-year survival rate to 70% in osteosarcoma patients, the treatment and survival rates have shown very little improvement in the last decades [6,7]. Therefore, it is urgent to explore novel molecule target for developing new therapeutic drugs and improving clinical outcome in osteosarcoma patients.

LncRNAs (lncRNAs) are a member of non-coding RNAs family with longer than 200 nts and no protein-coding ability, and have been suggested to be involved in a wide range of physiological and pathological processes including carcinogenesis [8–10]. LncRNA LOXL1 antisense RNA 1 (LOXL1-AS1) is located on human chromosome 15q24.1 and consists of 10,781 nucleotides with five exons. Originally, oxidative stress and cyclic mechanical stress induced the dysregulation of LOXL1-AS1 expression in human lens epithelial cells and human Schlemm’s canal endothelial cells respectively [11]. Afterward, the role of LOXL1-AS1 has been investigated in several types of human cancer including lung cancer [12], hepatocellular carcinoma [13], glioma [14,15], breast cancer [16,17], prostate cancer [18], and medulloblastoma [19]. However, the expression pattern and function of LOXL1-AS1 in osteosarcoma is still unknown. Therefore, the purpose of the present study is to investigate the clinical significance and biological function of LOXL1-AS1 in osteosarcoma.

## Materials and methods

### Patient samples

Total 58 fresh osteosarcoma tissue specimens and 20 fresh adjacent normal tissue specimens were obtained from Beijing Friendship Hospital. In addition, 68 fresh osteosarcoma tissue specimens and 22 fresh adjacent normal tissue specimens were obtained from Tongliang Hospital of Traditional Chinese Medicine Affiliated to School of Chinese Medicine of Chongqing Medical University. There was no significant difference in sample information between the two hospitals. Fresh tissue specimens were frozen in liquid nitrogen immediately and stored at −80°C. Histological and pathological diagnosis of each sample was confirmed by at least two pathologists. All cases did not receive antitumor treatment before surgery or biopsy. This research was reviewed and approved by the Ethics Committee of Beijing Friendship Hospital (no.14009) and Tongliang Hospital of Traditional Chinese Medicine Affiliated to School of Chinese Medicine of Chongqing Medical University (no.C140032). Written consent of each osteosarcoma patient was obtained before participating in the present study.

### Cell lines

Four human osteosarcoma cell lines (MG63, U-2 OS, Saos-2, and HOS) and a human normal osteoblast cell line (hFOB1.19) were purchased from American Type Culture Collection (ATCC, Manassas, VA, U.S.A.). All cells were cultured in Dulbecco’s modified Eagle’s medium (DMEM; Gibco, Grand Island, NY, U.S.A.) containing 10% fetal bovine serum (FBS; Gibco, Grand Island, NY, U.S.A.) at 37°C in a 5% CO_2_ humidified incubator.

### RNA extraction and quantitative real-time PCR

Total RNAs from tissues and cells were extracted with TRIzol (Invitrogen, Carlsbad, CA, U.S.A.) and reversely transcribed into complementary DNA by using Revert Aid First Strand cDNA Synthesis Kit (Thermo Fisher Scientific, Waltham, MA, U.S.A.) with random primers. Then, the PCR process was performed with Power SYBR Green PCR Master Mix (Life Technologies, Carlsbad, CA, U.S.A.) on an ABI 7500 (Applied Biosystems, Foster City, CA, U.S.A.). The specific primer sequences of LOXL1-AS1 and GAPDH were as follow: LOXL1-AS1 forward primer, 5′-TTCCCATTTACCTGCCCGAAG-3′; LOXL1-AS1 reverse primer, 5′-GTCAGCAAACACATGGCAAC-3′; GAPDH forward primer, 5′-GGAAGGACTCATGACCACAGTCC-3′; GAPDH reverse primer, 5′-TCGCTGTTGAAGTCAGAGGAGACC -3′. GAPDH was used as an internal control.

### Cell transfection

siRNA targetted to LOXL1-AS1 (si-LOXL1-AS1) and corresponding negative control (si-NC) purchased from Genechem (Shanghai, China) for knocking down LOXL1-AS1 expression and transferred into osteosarcoma cells using Lipofectamine RNAiMAX (Invitrogen, Carlsbad, CA, U.S.A.) according to the manufacturer’s instructions. After successful transfection for 48 h, the osteosarcoma cells were collected for the following *in vitro* experiments.

### MTT assay

MTT assay was used to detect the cell proliferation. After culture for 48, 48, 72, or 96 h, transfected osteosarcoma cells were incubated with 20 μl (10 mg/ml) MTT solution for 4 h. Afterward, 150 μl DMSO was added to dissolve the crystals. The optical density of each well was measured at the wavelength of 490 nm. All experiments were performed in triplicate.

### Cell cycle analysis

Cell cycle analysis was determined by flow cytometry using Cell Cycle Staining Ki (MultiSciences, Hangzhou, China). Transfected osteosarcoma cells were fixed with ethanol, and RNase was added for RNA degradation. Samples were stained with propidium iodide and analyzed by flow cytometry according to the manufacturer’s guidelines. All experiments were performed in triplicate.

### Cell migration and invasion assays

24-well transwell chambers (Corning, Kennebunk, ME, U.S.A.) were used to conduct cell migration assay. For cell invsion assay, the transwell chambers were coated with Matrigel (BD Biosciences, U.S.A.). Briefly, transfected osteosarcoma cells were suspended in FBS-free DMEM and seeded into the upper well. Then, the lower well was added with DMEM containing 20% FBS. Following 24 h incubation, cells on the lower surface of filter were fixed with methanol and stained with crystal violet, while cells on the upper surface of filter were removed with a cotton swab. The cell numbers on the lower surface of filter were counted in five random microscopic fields under a microscope. The experiments were repeated in triplicate.

### Protein isolation and western blot

The cells were lysed in RIPA lysis buffer (Beyotime, Shanghai, China) with protease inhibitor. Then, equal proteins were separated on 10% SDS-PAGE and transferred to polyvinylidene fluoride membrane. PI3K, phosphorylated PI3K (p-PI3K), AKT, phosphorylated AKT (p-AKT) proteins were detected by monoclonal antibodies for PI3K, p-PI3K, AKT, p-AKT (1:1,000; Cell Signaling Technology, Beverly, MA, U.S.A.) and visualized by the enhanced chemiluminescence system (Amersham, Arlington Heights, IL, U.S.A.). The density of the bands was quantitated using the imaging system (Bio-Rad, Hercules, CA, U.S.A.). All experiments were performed in triplicate.

### Statistical analysis

SPSS 18.0 software (IBM, Armonk, NY, U.S.A.) and GraphPad Prism 5.0 software (La Jolla, CA, U.S.A.) were utilized to analyze all the statistical data. Student’s *t*test was used to analyze the difference between two groups. Correlations between LOXL1-AS1 expression and clinicpathological features of osteosarcoma patients were estimated by chi-square test. Survival curves were made by Kaplan–Meier method, and log-rank test was used for comparing the survival distributions. Univariate and multivariate Cox regression analyses were used for identifying independent prognostic factors in osteosarcoma patients. A *P* value <0.05 was considered statistically significant.

## Results

### The LOXL1-AS1 expression in human osteosarcoma tissues and cell lines

**Figure 1:**
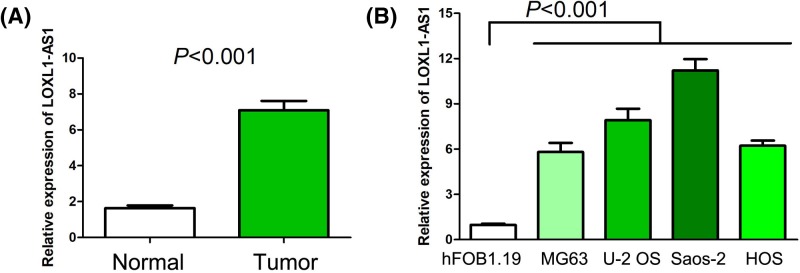
The LOXL1-AS1 expression in human osteosarcoma tissues and cell lines (**A**) LOXL1-AS1 expression was remarkably increased in osteosarcoma tissues specimens compared with normal bone tissue specimens. (**B**) Levels of LOXL1-AS1 expression in osteosarcoma cell lines (MG63, U2OS, Saos-2, and HOS) was profoundly higher than human normal osteoblast cell line (hFOB1.19).

To initially measure the LOXL1-AS1 expression in osteosarcoma, we collected 96 osteosarcoma tissues specimens and 24 normal bone tissue specimens. Compared with normal bone tissue specimens, LOXL1-AS1 expression was remarkably increased in osteosarcoma tissues specimens (*P*<0.001, Figure 1A). Moreover, we detected LOXL1-AS1 expression in osteosarcoma cell lines and a human normal osteoblast cell line and observed that levels of LOXL1-AS1 expression in osteosarcoma cell lines (MG63, U2OS, Saos-2, and HOS) was profoundly higher than human normal osteoblast cell line (hFOB1.19) (*P*<0.001, Figure 1B).

### The correlation between LOXL1-AS1 expression and clinicopathological characteristics in osteosarcoma

For estimating the clinical significance of LOXL1-AS1 expression in osteosarcoma, all cases in this research were classified into high LOXL1-AS1 expression group (*n*=63) and low LOXL1-AS1 expression group (*n*=63) in accordance to the median value of LOXL1-AS1 expression. Then, we performed chi-square test to assess correlations between LOXL1-AS1 expression and clinicopathological characteristics of osteosarcoma, and observed high-expression of LOXL1-AS1 was correlated with Enneking stage (*P*<0.001, Table 1), tumor size (*P*=0.004, Table 1), distant metastasis (*P*=0.001, Table 1) and histological grade (*P*=0.001, Table 1). However, there was no obvious correlation between LOXL1-AS1 expression and other of clinicopathological characteristics including gender (*P*=0.353, Table 1), age (*P*=0.139, Table 1), and tumor site (*P*=0.348, Table 1).

**Table 1:** Associations between LOXL1-AS1 expression and clinicopathological characteristics in osteosarcoma cases

Characteristics	*n*	High expression	Low expression	*P*
Age (y)				
≤18	46	27	19	0.139
>18	80	36	44	
Gender				
Female	45	20	25	0.353
Male	81	43	38	
Enneking stage				
I-IIA	46	13	33	<0.001
IIB-III	80	50	30	
Tumor size				
≤8 cm	76	30	46	0.004
>8 cm or discontinuous tumors	50	33	17	
Distant metastasis				
Absence	104	45	59	0.001
Presence	22	18	4	
Histological grade				
G1–G2	57	19	38	0.001
G3–G4	69	44	25	
Tumor site				
Femur/tibia	104	50	54	0.348
Other	22	13	9	

### The correlation between LOXL1-AS1 expression and overall survival in osteosarcoma

**Figure 2:**
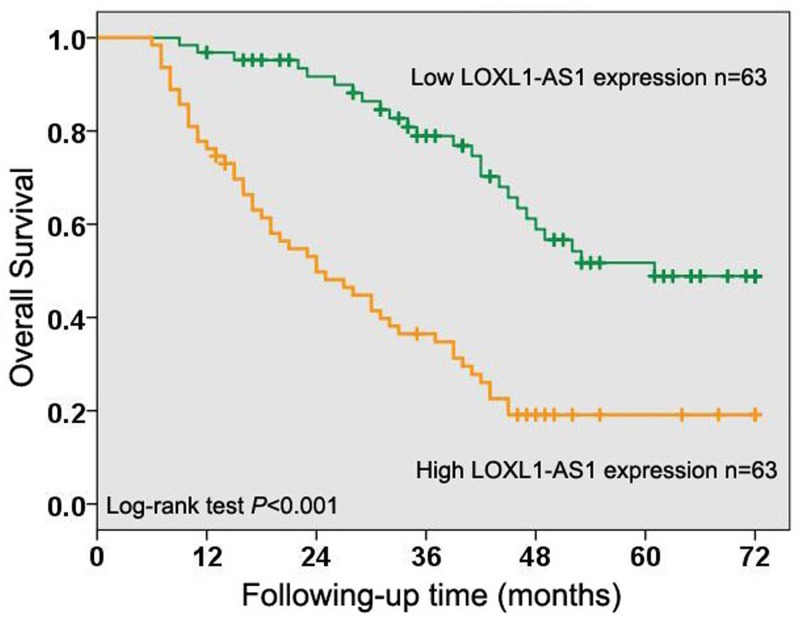
The correlation between LOXL1-AS1 expression and overall survival in osteosarcoma Kaplan–Meier method and log-rank test were used to analyze the correlation between LOXL1-AS1 expression and overall survival in osteosarcoma and showed that osteosarcoma cases with high levels of LOXL1-AS1 expression had poorer overall survival than those with low levels of LOXL1-AS1 expression.

In order to evaluate the importance of LOXL1-AS1 expression for the clinical outcome in osteosarcoma patients, Kaplan–Meier method and log-rank test were used to analyze the correlation between LOXL1-AS1 expression and overall survival in osteosarcoma. The results showed osteosarcoma cases with high levels of LOXL1-AS1 expression had poorer overall survival than those with low levels of LOXL1-AS1 expression (*P*<0.001, Figure 2). Moreover, we performed univariate Cox regression analysis and identified Enneking stage (*P*<0.001, Table 2), tumor size (*P*=0.012, Table 2), distant metastasis (*P*<0.001, Table 2), histological grade (*P*<0.001, Table 2), and LOXL1-AS1 expression (*P*<0.001, Table 2) as prognostic factors for overall survival in osteosarcoma cases. Furthermore, the multivariate Cox regression analysis suggested high LOXL1-AS1 expression was an independent poor predictor in osteosarcoma patients (*P*=0.024, Table 2).

**Table 2:** Univariate and multivariate Cox regression of prognostic factors in osteosarcoma patients

Parameter	Univariate analysis			Multivariate analysis		
	HR	95% CI	*P*	HR	95% CI	*P*
Age (y)						
(≤18 vs >18)	0.698	0.441–1.104	0.124			
Gender						
(Female vs ≥male)	1.351	0.832–2.195	0.224			
Enneking stage						
(I–II A vs II B–III)	2.949	1.723–5.045	<0.001	1.134	0.486–2.646	0.770
Tumor size						
(≤8 vs >8 cm or discontinuous tumors)	1.808	1.140–2.868	0.012	1.227	0.744–2.023	0.423
Distant metastasis						
(Absence vs presence)	6.007	3.334–10.823	<0.001	3.592	1.906–6.767	<0.001
Histological grade						
(G1–G2 vs G3–G4)	2.578	1.572–4.227	<0.001	1.589	0.772–3.272	0.209
Tumor site						
(Femur/tibia vs other)	1.586	0.909–2.766	0.104			
LOXL1-AS1 expression						
(Low vs high)	3.374	2.071–5.496	<0.001	1.985	1.096–3.597	0.024

Abbreviation: HR, hazard ratio.

### The biological function of LOXL1-AS1 in osteosarcoma

**Figure 3:**
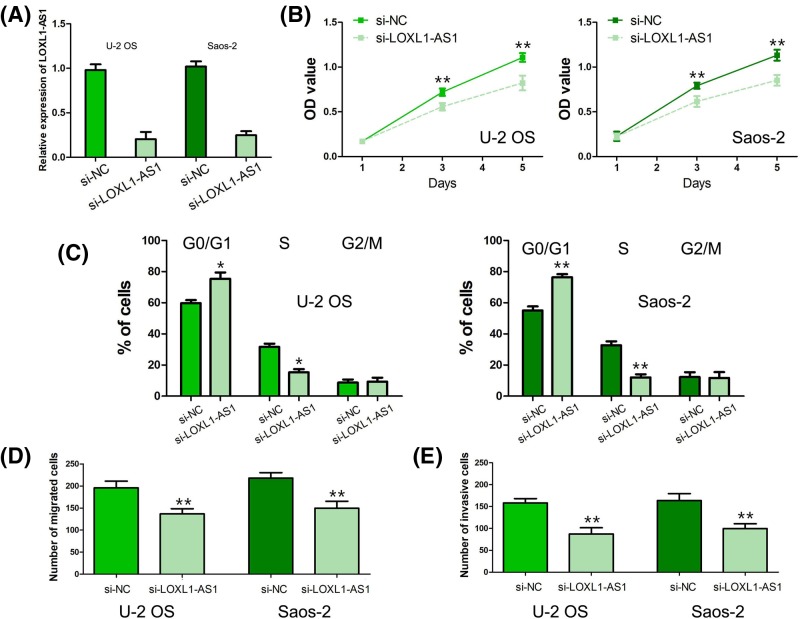
The biological function of LOXL1-AS1 in osteosarcoma (**A**) The efficiency of si-LOXL1-AS1 in osteosarcoma cells was verified by qRT-PCR. (**B**) Osteosarcoma cell proliferation was obviously depressed after U-2 OS and Saos-2 cells were transfected with si-LOXL1-AS1. (**C**) Knockdown of LOXL1-AS1 led to cell cycle arrest at G0/G1 phase in osteosarcoma cells. (**D**,**E**) Knockdown of LOXL1-AS1 dramatically inhibited osteosarcoma cell migration and invasion abilities. (*: *P*<0.01, **: *P*<0.001).

To explore the biological function of LOXL1-AS1 in osteosarcoma, U-2 OS and Saos-2 cells were transfected with si-LOXL1-AS1 and the efficiency of si-LOXL1-AS1 in osteosarcoma cells was verified by qRT-PCR (Figure 3A). The results of MTT assay showed that osteosarcoma cell proliferation was obviously depressed after U-2 OS and Saos-2 cells were transfected with si-LOXL1-AS1 (*P*<0.001, Figure 3B). The cell cycle analysis indicated that the cell proportion was significantly increased in the G1 phase and markedly decreased in the S phase after U-2 OS and Saos-2 cells were transfected with si-LOXL1-AS1, which suggested that knockdown of LOXL1-AS1 led to cell cycle arrest at G0/G1 phase (*P*<0.01, Figure 3C). Moreover, the results of transwell migration and invasion assays revealed that knockdown of LOXL1-AS1 dramatically inhibited osteosarcoma cell migration and invasion abilities in U-2 OS and Saos-2 cells (*P*<0.001, Figure 3D,E).

### The molecular mechanism of LOXL1-AS1 in osteosarcoma

**Figure 4:**
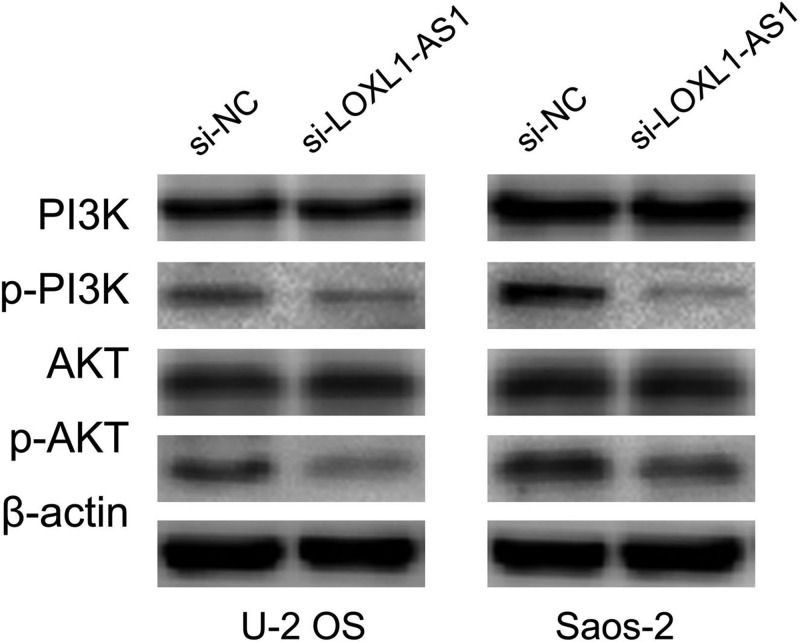
The molecular mechanism of LOXL1-AS1 in osteosarcoma Knockdown of LOXL1-AS1 significantly inhibited p-PI3K and p-AKT expression but had no effect on PI3K and AKT expression in osteosarcoma cells.

The published reported showed that LOXL1-AS1 enhanced medulloblastoma cell proliferation and motility via activating PI3K-AKT pathway [19]. Meanwhile, PI3K-AKT pathway has been suggested to be involved in osteosarcoma cell proliferation, cell cycle, migration, and invasion [20–22]. Therefore, we measure the effect of LOXL1-AS1 on PI3K and AKT expression through western blot. The results suggested knockdown of LOXL1-AS1 significantly inhibited p-PI3K and p-AKT expression but had no effect on PI3K and AKT expression in U-2 OS and Saos-2 cells (Figure 4).

## Discussion

LOXL1-AS1 is a lncRNA encoded on the opposite strand of lysyl oxidase-like 1 (LOXL1), which was found to strongly associated with exfoliation glaucoma and exfoliation syndrome [23,24]. Subsequently, the dysregulation of LOXL1-AS1 was gradually discovered in human cancers. Wang et al. analyzed a non-small cell lung cancer microarray (GSE18842) and observed that LOXL1-AS1 expression was elevated in tumor tissues compared with normal tissues [12]. Moreover, Wang et al. analyzed 160 glioblastoma RNA-seq data at the Chinese Glioma Genome Atlas and found that the mesenchymal subtype of glioblastoma had high level of LOXL1-AS1 expression [15]. In medulloblastoma, Gao et al. found levels of LOXL1-AS1 expression were notably increased in tumor samples compared with adjacent noncancerous samples [19]. However, Xu et al. also analyzed the genome-wide lncRNA expression profiles of breast cancer at The Cancer Genome Atlas (TCGA) database and found that there was no statistical difference of LOXL1-AS1 expression between breast cancer tissues and normal mammary tissues [16]. Up to now, there was no report about the expression pattern of LOXL1-AS1 in osteosarcoma. In our study, we first found LOXL1-AS1 expression levels were higher in osteosarcoma tissues and cell lines than normal bone tissues and normal osteoblast cell line, respectively. Meanwhile, we further assessed the clinical significance of LOXL1-AS1 expression in osteosarcoma patients via analyzing correlations between LOXL1-AS1 expression and clinicopathological characteristics, and observed that high-expression of LOXL1-AS1 was correlated with Enneking stage distant metastasis and histological grade. In medulloblastoma patients, high-expression of LOXL1-AS1 was associated with advanced clinical stage [19]. In addition, Zhang et al. classified hepatocellular carcinoma samples into epithelial and mesenchymal subtypes and screened differential lncRNAs expression, and found that LOXL1-AS1 was overexpressed in the mesenchymal subtype comparing with the epithelial subtype [13]. Besides, Mathias et al. indentified LOXL1-AS1 as a specific lncRNA in basal-like breast cancer [17].

The prognostic value of LOXL1-AS1 was seldom reported in human cancers. Wang et al. reported high LOXL1-AS1expression acted as unfavorable prognostic factor for overall survival in glioblastoma patients [15]. In our study, we found osteosarcoma patients with high levels of LOXL1-AS1 expression had poorer overall survival than those with low levels of LOXL1-AS1 expression, and LOXL1-AS1 overexpression as an independent poor predictor in osteosarcoma patients. Moreover, we tried to the prognostic significance of LOXL1-AS1 expression in thirty types of human cancer at TCGA database and found that LOXL1-AS1 expression was associated with clinical outcome in nine kinds of tumor. LOXL1-AS1 overexpression was found to be favorable prognostic biomarker in adrenocortical carcinoma, uterine corpus endometrial carcinoma, and uveal melanoma, and to be unfavorable prognostic biomarker in cholangio carcinoma, glioblastoma multiforme, kidney renal clear cell carcinoma, brain lower grade glioma, mesothelioma, and pancreatic adenocarcinoma. Generally, more studies would be needed to verify the prognostic value of LOXL1-AS1 expression in human cancers.

LOXL1-AS1 has been suggested to act as oncogenic lncRNA in glioblastoma [15], prostate cancer [18], and medulloblastom [19]. Wang et al. showed knockdown of LOXL1-AS1 inhibited glioblastoma cell proliferation and weakened mesenchymal characteristics through decreasing NF-κB pathway [15]. In prostate cancer, silencing of LOXL1-AS1 suppressed cell proliferation and arrested cell cycle progression via regulating miR-541-3p and CCND1 [18]. Moreover, Gao et al. reported inhibition of LOXL1-AS1 depressed cell proliferation, migration, tumor growth, and induced cell cycle arrest and cell apoptosis through modulating PI3K-AKT pathway [19]. In our study, we found knockdown of LOXL1-AS1 dramatically inhibited osteosarcoma cell proliferation, migration, and invasion through suppressing PI3K-AKT pathway. Besides, PI3K/AKT activation has been suggested to involve in the stemness maintenance in human cancers [25–27]. Therefore, we will try to explore the effect of LOXL1-AS1 on the stemness maintenance in osteosarcoma for overcoming the drug resistance and relapse [28].

## Conclusion

LOXL1-AS1 is a novel biomarker for predicting clinical progression and poor prognosis, and functions as tumor promoter to regulate osteosarcoma cell proliferation, migration, and invasion through PI3K-AKT pathway.

## Abbreviations

AS1: antisense RNA 1
DMEM: Dulbecco’s modified Eagle’s medium
FBS: fetal bovine serum
LOXL1: lysyl oxidase-like 1
qRT-PCR: quantitative real-time PCR
TCGA: The Cancer Genome Atlas
